# Tetra­aqua­diazido­cobalt(II) 3,3′-dicarb­oxy­l­ato-1,1′-ethyl­enedipyridinium

**DOI:** 10.1107/S1600536810046143

**Published:** 2010-11-13

**Authors:** Yan-Qing Wen, Chun-Yan Tian, En-Qing Gao

**Affiliations:** a Shanghai Key Laboratory of Green Chemistry and Chemical Processes, Department of Chemistry, East China Normal University, Shanghai 200062, People’s Republic of China

## Abstract

The asymmetric unit of the title compound, [Co(N_3_)_2_(H_2_O)_4_]·C_14_H_12_N_2_O_4_, comprises half of the cobalt(II) complex mol­ecule and a half of the 3,3′-dicarboxyl­ato-1,1′-ethyl­enedipyridinium mol­ecule. The Co^II^ atom is located on an inversion centre and hence the complex mol­ecule adopts a centrosymmetric *trans*-octa­hedral geometry. The zwitterionic organic mol­ecule is also centrosymmetric with the centre of the C—C bond of the ethyl­ene moiety coinciding with an inversion centre. The adduct of metal complex and organic mol­ecule is associated into a three-dimenional network through O—H⋯O hydrogen bonds.

## Related literature

For background to hydrogen bonds, see: Braga & Grepioni (2000 ▶); Fabbiani *et al.* (2010 ▶); Salitros *et al.* (2010 ▶); Schultheis *et al.* (2010 ▶). For the synthesis of the ligand, see: Loeb *et al.* (2006 ▶). For hydrogen-bond motifs, see: Bernstein *et al.* (1995 ▶); Etter (1990 ▶).
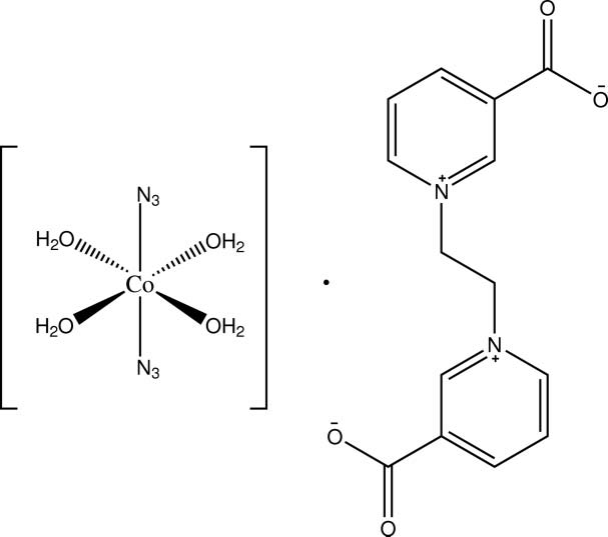


## Experimental

### 

#### Crystal data

[Co(N_3_)_2_(H_2_O)_4_]·C_14_H_12_N_2_O_4_
*M* *_r_* = 487.31
Triclinic, 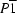

*a* = 7.4309 (6) Å
*b* = 7.7507 (7) Å
*c* = 8.5582 (7) Å
α = 95.463 (2)°
β = 90.586 (2)°
γ = 95.011 (2)°
*V* = 488.71 (7) Å^3^
*Z* = 1
Mo *K*α radiation
μ = 0.94 mm^−1^
*T* = 296 K
0.25 × 0.20 × 0.15 mm

#### Data collection

Bruker APEXII CCD area-detector diffractometer
Absorption correction: multi-scan (*SADABS*; Bruker, 2008 ▶) *T*_min_ = 0.799, *T*_max_ = 0.872
6091 measured reflections
1907 independent reflections
1889 reflections with *I* > 2σ(*I*)
*R*_int_ = 0.015

#### Refinement

*R*[*F* ^2^ > 2σ(*F* ^2^)] = 0.024
*wR*(*F* ^2^) = 0.074
*S* = 1.12
1907 reflections
154 parameters
9 restraints
H atoms treated by a mixture of independent and constrained refinement
Δρ_max_ = 0.28 e Å^−3^
Δρ_min_ = −0.28 e Å^−3^

### 

Data collection: *APEX2* (Bruker, 2007 ▶); cell refinement: *SAINT* (Bruker, 2007 ▶); data reduction: *SAINT*; program(s) used to solve structure: *SHELXS97* (Sheldrick, 2008 ▶); program(s) used to refine structure: *SHELXL97* (Sheldrick, 2008 ▶); molecular graphics: *SHELXTL* (Sheldrick, 2008 ▶); software used to prepare material for publication: *SHELXTL*.

## Supplementary Material

Crystal structure: contains datablocks I, global. DOI: 10.1107/S1600536810046143/kp2284sup1.cif

Structure factors: contains datablocks I. DOI: 10.1107/S1600536810046143/kp2284Isup2.hkl

Additional supplementary materials:  crystallographic information; 3D view; checkCIF report

## Figures and Tables

**Table 1:** Selected bond lengths (Å)

Co1—O4	2.0780 (12)
Co1—N2	2.0958 (15)
Co1—O3	2.1431 (12)

**Table 2:** Hydrogen-bond geometry (Å, °)

*D*—H⋯*A*	*D*—H	H⋯*A*	*D*⋯*A*	*D*—H⋯*A*
O3—H3*B*⋯O1^i^	0.84 (2)	2.01 (2)	2.8180 (18)	163 (2)
O3—H3*C*⋯O1^ii^	0.84 (2)	1.91 (2)	2.7395 (17)	172 (2)
O4—H4*C*⋯O2^iii^	0.86 (2)	1.84 (2)	2.6901 (18)	173 (3)
O4—H4*B*⋯O2	0.81 (2)	2.03 (2)	2.8028 (18)	159 (2)

Symmetry codes: (i) 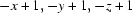; (ii) 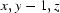; (iii) 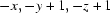.

**Fig. 1.**
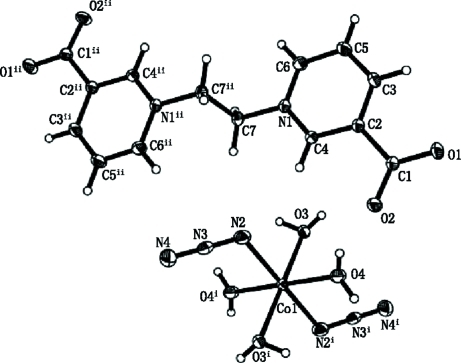
The molecular structure of the title compound showing the atom-labelling scheme. Displacement ellipsoids are drawn at the 30% probability level. [Symmetry code: (i) -x, -y, 1 - z; (ii) 1 - x, -y, -z].

**Fig. 2.**
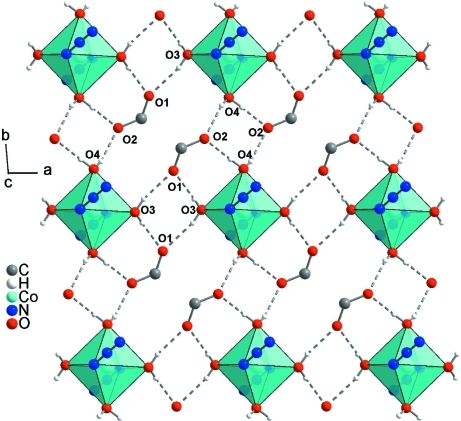
Two-dimensional layer structure connected through intermolecula O—H···O hydrogen bonds. [Symmetry code: (i) -x + 1, -y + 1, -z + 1; (ii) x, y - 1, z; (iii)-x, -y + 1, -z + 1].

**Fig. 3.**
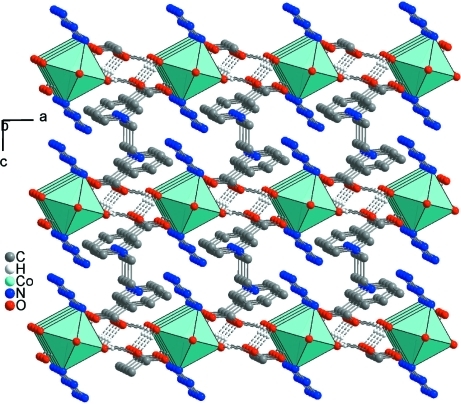
Three dimensional structure connected by the organic ligands interlinking the hydrogen-bonded sheets.

## supplementary crystallographic information

### Comment

Hydrogen bonds play a key role in biological systems and materials, and they have been widely used as a putative supramolecular tool for engineering organic and metal-organic solids (Fabbiani *et al.*, 2010; Salitros *et al.*, 2010; Schultheis *et al.*, 2010; Braga & Grepioni, 2000). In this paper, we report the structure of the title compound, (I), which contains a neutral metal complex molecule, [Co(N_3_)_2_(H_2_O)_4_], and a zwitterionic dicarboxylate, 1,2-bis(3-carboxylatopyridinium)ethane (Fig. 1). The metal complex molecule is centrosymmetric, with the octahedral-coordinated Co^II^  by two azide anions and four water molecules in a *trans* arrangement (Fig. 1, Table 1). Two opposite Co—O distances are longer than the Co—N and other Co—O ones, defining an axially elongated geometry. The zwitterionic molecule is also centrosymmetric (Fig.1 ). The inorganic complex molecules and the carboxylate groups are associated into a sheet through O—H···O hydrogen bonds involving the coordinated aqua ligands (O3 and O4) and the carboxylate oxygen atoms (O1 and O2) (Fig. 2, Table 2). The two O4 aqua ligands from symmetry related complex molecules and two O2 atoms from symmetry related organic molecules form a hydrogen-bonded ring which can be denoted by the graph set *R*_4_^2^(8) (Bernstein *et al.*, 1995; Etter, 1990). Similar hydrogen-bonded rings are formed by O1 and O3. The carboxylate group forms a *R*_2_^2^(8) hydrogen-bonded ring with two aqua ligands from the same complex molecule. Besides, a large hydrogen-bonded ring [*R*_4_^4^(16)] is formed by two carboxylate groups and four aqua ligands from two complex molecules. The organic ligands interlink the hydrogen-bonded sheets of the metal complexes into the three-dimensional structure (Fig. 3).

### Experimental

The zwitterionic ligand ([H_2_*L*^1^]Br_2_) was synthesised from 1,2-dibromoethane and ethyl nicotinate according to the published procedure (Loeb *et al.*, 2006). An aqueous solution (4 mL) of [H_2_*L*^1^]Br_2_ (0.1 mmol) and NaN_3_ (1 mmol) was added to a DMF solution (1.5 mL) of Co(ClO_4_)_2_.6H_2_O (0.2 mmol) with stirring. The resulting solution was allowed to evaporate slowly at room temperature, yielding light-red block crystals of (I) in three days. Yield: 75%. Anal. calcd (found) (%) for CoC_14_H_20_N_8_O_8_: C, 34.79 (34.51); H, 4.39 (4.14); N, 22.87 (23.00). Main IR bands (KBr, ν/cm^-1^): 3427*m*, 3097w, 2042 s, 1637 s, 1606 s, 1392*m*, 765*m*, 688*m*.

### Refinement

All hydrogen atoms attached to carbon atoms were placed at calculated positions and refined with the riding model using AFIX 43 and AFIX 23 instructions for aromatic C—H and secondary CH_2_. The water hydrogen atoms were initially located from difference Fourier maps and refined isotropically with restraints on O—H distance (0.85 Å) and H—O—H angle, and *U*_iso_(H) = 1.5*U*_eq_(O). The 'rigid-bond' restraint was applied on the azide moiety (N2—N3—N4) using the *SHELXL* DELU instruction.

### Crystal data

**Table:**

[Co(N_3_)_2_(H_2_O)_4_]·C_14_H_12_N_2_O_4_	*Z* = 1
*M**_r_* = 487.31	*F*(000) = 251
Triclinic, *P*1	*D*_x_ = 1.656 Mg m^−^^3^
Hall symbol: -P 1	Mo *K*α radiation, λ = 0.71073 Å
*a* = 7.4309 (6) Å	Cell parameters from 15377 reflections
*b* = 7.7507 (7) Å	θ = 3.4–27.5°
*c* = 8.5582 (7) Å	µ = 0.94 mm^−^^1^
α = 95.463 (2)°	*T* = 296 K
β = 90.586 (2)°	Block, red
γ = 95.011 (2)°	0.25 × 0.20 × 0.15 mm
*V* = 488.71 (7) Å^3^	

### Data collection

**Table:**

Bruker APEXII CCD area-detector diffractometer	1907 independent reflections
Radiation source: fine-focus sealed tube	1889 reflections with *I* > 2σ(*I*)
graphite	*R*_int_ = 0.015
phi and ω scans	θ_max_ = 26.1°, θ_min_ = 2.4°
Absorption correction: multi-scan (*SADABS*; Bruker, 2008)	*h* = −9→9
*T*_min_ = 0.799, *T*_max_ = 0.872	*k* = −9→8
6091 measured reflections	*l* = −10→10

### Refinement

**Table:**

Refinement on *F*^2^	Primary atom site location: structure-invariant direct methods
Least-squares matrix: full	Secondary atom site location: difference Fourier map
*R*[*F*^2^ > 2σ(*F*^2^)] = 0.024	Hydrogen site location: inferred from neighbouring sites
*wR*(*F*^2^) = 0.074	H atoms treated by a mixture of independent and constrained refinement
*S* = 1.12	*w* = 1/[σ^2^(*F*_o_^2^) + (0.0411*P*)^2^ + 0.2239*P*] where *P* = (*F*_o_^2^ + 2*F*_c_^2^)/3
1907 reflections	(Δ/σ)_max_ < 0.001
154 parameters	Δρ_max_ = 0.28 e Å^−^^3^
9 restraints	Δρ_min_ = −0.28 e Å^−^^3^

### Special details

**Table:**

Geometry. All e.s.d.'s (except the e.s.d. in the dihedral angle between two l.s. planes) are estimated using the full covariance matrix. The cell e.s.d.'s are taken into account individually in the estimation of e.s.d.'s in distances, angles and torsion angles; correlations between e.s.d.'s in cell parameters are only used when they are defined by crystal symmetry. An approximate (isotropic) treatment of cell e.s.d.'s is used for estimating e.s.d.'s involving l.s. planes.
Refinement. Refinement of *F*^2^ against ALL reflections. The weighted *R*-factor *wR* and goodness of fit *S* are based on *F*^2^, conventional *R*-factors *R* are based on *F*, with *F* set to zero for negative *F*^2^. The threshold expression of *F*^2^ > σ(*F*^2^) is used only for calculating *R*-factors(gt) *etc*. and is not relevant to the choice of reflections for refinement. *R*-factors based on *F*^2^ are statistically about twice as large as those based on *F*, and *R*- factors based on ALL data will be even larger.

### Fractional atomic coordinates and isotropic or equivalent isotropic displacement parameters (Å^2^)

**Table:**

	*x*	*y*	*z*	*U*_iso_*/*U*_eq_	
Co1	0.0000	0.0000	0.5000	0.02282 (12)	
C1	0.3729 (2)	0.5896 (2)	0.3420 (2)	0.0283 (3)	
C2	0.5025 (2)	0.4708 (2)	0.26023 (19)	0.0258 (3)	
C3	0.6822 (2)	0.5278 (2)	0.2423 (2)	0.0335 (4)	
H3A	0.7234	0.6424	0.2751	0.040*	
C4	0.4437 (2)	0.3011 (2)	0.20731 (19)	0.0255 (3)	
H4A	0.3228	0.2613	0.2153	0.031*	
C5	0.8009 (2)	0.4148 (3)	0.1756 (3)	0.0392 (4)	
H5A	0.9215	0.4530	0.1625	0.047*	
C6	0.7382 (2)	0.2466 (2)	0.1293 (2)	0.0339 (4)	
H6A	0.8173	0.1685	0.0875	0.041*	
C7	0.5003 (2)	0.0111 (2)	0.08913 (19)	0.0286 (3)	
H7A	0.3795	−0.0177	0.1265	0.034*	
H7B	0.5803	−0.0667	0.1303	0.034*	
N1	0.56206 (18)	0.19334 (17)	0.14405 (16)	0.0257 (3)	
N2	0.0817 (2)	−0.0222 (2)	0.26583 (18)	0.0399 (4)	
N3	−0.0146 (2)	−0.1006 (2)	0.16704 (17)	0.0320 (3)	
N4	−0.1073 (3)	−0.1740 (2)	0.0672 (2)	0.0461 (4)	
O1	0.43867 (18)	0.73681 (16)	0.39597 (19)	0.0435 (4)	
O2	0.21281 (17)	0.52889 (16)	0.35044 (18)	0.0405 (3)	
O3	0.27781 (16)	−0.00456 (16)	0.56790 (15)	0.0319 (3)	
H3B	0.347 (3)	0.086 (2)	0.569 (3)	0.048*	
H3C	0.321 (3)	−0.082 (2)	0.508 (3)	0.048*	
O4	0.02796 (18)	0.26995 (16)	0.50991 (18)	0.0379 (3)	
H4B	0.090 (3)	0.323 (3)	0.449 (3)	0.057*	
H4C	−0.053 (3)	0.334 (3)	0.547 (3)	0.057*	

### Atomic displacement parameters (Å^2^)

**Table:**

	*U*^11^	*U*^22^	*U*^33^	*U*^12^	*U*^13^	*U*^23^
Co1	0.02008 (17)	0.02004 (17)	0.02747 (17)	−0.00003 (11)	0.00302 (11)	−0.00082 (11)
C1	0.0273 (8)	0.0202 (7)	0.0363 (8)	0.0007 (6)	0.0062 (7)	−0.0031 (6)
C2	0.0247 (8)	0.0210 (7)	0.0305 (8)	0.0009 (6)	0.0035 (6)	−0.0024 (6)
C3	0.0282 (9)	0.0247 (8)	0.0445 (10)	−0.0047 (7)	0.0040 (7)	−0.0067 (7)
C4	0.0220 (7)	0.0230 (8)	0.0301 (8)	0.0002 (6)	0.0040 (6)	−0.0026 (6)
C5	0.0231 (8)	0.0348 (9)	0.0565 (12)	−0.0035 (7)	0.0081 (8)	−0.0077 (8)
C6	0.0247 (8)	0.0311 (9)	0.0447 (10)	0.0045 (7)	0.0068 (7)	−0.0056 (7)
C7	0.0331 (8)	0.0179 (7)	0.0336 (9)	0.0000 (6)	0.0054 (7)	−0.0030 (6)
N1	0.0256 (7)	0.0203 (6)	0.0297 (7)	0.0006 (5)	0.0035 (5)	−0.0043 (5)
N2	0.0335 (8)	0.0555 (10)	0.0297 (8)	−0.0006 (7)	0.0064 (6)	0.0028 (7)
N3	0.0314 (8)	0.0343 (8)	0.0318 (8)	0.0069 (6)	0.0117 (7)	0.0059 (6)
N4	0.0475 (10)	0.0485 (10)	0.0398 (9)	−0.0053 (8)	0.0032 (8)	−0.0011 (8)
O1	0.0330 (7)	0.0226 (6)	0.0701 (10)	−0.0026 (5)	0.0105 (6)	−0.0159 (6)
O2	0.0282 (6)	0.0241 (6)	0.0665 (9)	−0.0017 (5)	0.0160 (6)	−0.0082 (6)
O3	0.0230 (6)	0.0274 (6)	0.0434 (7)	0.0016 (5)	0.0014 (5)	−0.0067 (5)
O4	0.0356 (7)	0.0210 (6)	0.0575 (8)	0.0026 (5)	0.0178 (6)	0.0028 (5)

### Geometric parameters (Å, °)

**Table:**

Co1—O4	2.0780 (12)	C5—C6	1.366 (3)
Co1—O4^i^	2.0780 (12)	C5—H5A	0.9300
Co1—N2	2.0958 (15)	C6—N1	1.349 (2)
Co1—N2^i^	2.0958 (15)	C6—H6A	0.9300
Co1—O3^i^	2.1431 (12)	C7—N1	1.478 (2)
Co1—O3	2.1431 (12)	C7—C7^ii^	1.519 (3)
C1—O1	1.245 (2)	C7—H7A	0.9700
C1—O2	1.246 (2)	C7—H7B	0.9700
C1—C2	1.521 (2)	N2—N3	1.188 (2)
C2—C4	1.381 (2)	N3—N4	1.164 (2)
C2—C3	1.384 (2)	O3—H3B	0.836 (15)
C3—C5	1.386 (3)	O3—H3C	0.839 (15)
C3—H3A	0.9300	O4—H4B	0.813 (16)
C4—N1	1.348 (2)	O4—H4C	0.856 (16)
C4—H4A	0.9300		
			
O4—Co1—O4^i^	180.0	N1—C4—H4A	120.1
O4—Co1—N2	91.41 (6)	C2—C4—H4A	120.1
O4^i^—Co1—N2	88.59 (6)	C6—C5—C3	119.02 (16)
O4—Co1—N2^i^	88.59 (6)	C6—C5—H5A	120.5
O4^i^—Co1—N2^i^	91.41 (6)	C3—C5—H5A	120.5
N2—Co1—N2^i^	180.0	N1—C6—C5	120.23 (16)
O4—Co1—O3^i^	88.83 (5)	N1—C6—H6A	119.9
O4^i^—Co1—O3^i^	91.17 (5)	C5—C6—H6A	119.9
N2—Co1—O3^i^	92.17 (6)	N1—C7—C7^ii^	109.09 (16)
N2^i^—Co1—O3^i^	87.83 (6)	N1—C7—H7A	109.9
O4—Co1—O3	91.17 (5)	C7^ii^—C7—H7A	109.9
O4^i^—Co1—O3	88.83 (5)	N1—C7—H7B	109.9
N2—Co1—O3	87.83 (6)	C7^ii^—C7—H7B	109.9
N2^i^—Co1—O3	92.17 (6)	H7A—C7—H7B	108.3
O3^i^—Co1—O3	180.0	C4—N1—C6	121.82 (14)
O1—C1—O2	126.73 (15)	C4—N1—C7	120.05 (13)
O1—C1—C2	116.50 (14)	C6—N1—C7	118.13 (14)
O2—C1—C2	116.75 (14)	N3—N2—Co1	120.05 (12)
C4—C2—C3	118.77 (14)	N4—N3—N2	178.01 (19)
C4—C2—C1	120.22 (14)	Co1—O3—H3B	119.4 (17)
C3—C2—C1	120.97 (14)	Co1—O3—H3C	107.1 (17)
C2—C3—C5	120.26 (16)	H3B—O3—H3C	108.0 (19)
C2—C3—H3A	119.9	Co1—O4—H4B	122.9 (18)
C5—C3—H3A	119.9	Co1—O4—H4C	123.1 (17)
N1—C4—C2	119.85 (14)	H4B—O4—H4C	109 (2)
			
O1—C1—C2—C4	175.22 (17)	C2—C4—N1—C6	−0.5 (3)
O2—C1—C2—C4	−3.6 (2)	C2—C4—N1—C7	179.21 (14)
O1—C1—C2—C3	−2.5 (3)	C5—C6—N1—C4	−1.6 (3)
O2—C1—C2—C3	178.67 (17)	C5—C6—N1—C7	178.70 (17)
C4—C2—C3—C5	−1.5 (3)	C7^ii^—C7—N1—C4	108.4 (2)
C1—C2—C3—C5	176.28 (18)	C7^ii^—C7—N1—C6	−71.9 (2)
C3—C2—C4—N1	2.0 (2)	O4—Co1—N2—N3	−122.40 (16)
C1—C2—C4—N1	−175.75 (15)	O4^i^—Co1—N2—N3	57.60 (16)
C2—C3—C5—C6	−0.6 (3)	O3^i^—Co1—N2—N3	−33.51 (16)
C3—C5—C6—N1	2.1 (3)	O3—Co1—N2—N3	146.49 (16)

Symmetry codes: (i) −*x*, −*y*, −*z*+1; (ii) −*x*+1, −*y*, −*z*.

### Hydrogen-bond geometry (Å, °)

**Table:**

*D*—H···*A*	*D*—H	H···*A*	*D*···*A*	*D*—H···*A*
O3—H3B···O1^iii^	0.84 (2)	2.01 (2)	2.8180 (18)	163 (2)
O3—H3C···O1^iv^	0.84 (2)	1.91 (2)	2.7395 (17)	172 (2)
O4—H4C···O2^v^	0.86 (2)	1.84 (2)	2.6901 (18)	173 (3)
O4—H4B···O2	0.81 (2)	2.03 (2)	2.8028 (18)	159 (2)

Symmetry codes: (iii) −*x*+1, −*y*+1, −*z*+1; (iv) *x*, *y*−1, *z*; (v) −*x*, −*y*+1, −*z*+1.

## References

[bb1] Bernstein, J., Davis, R. E., Shimoni, L. & Chang, N. L. (1995). *Angew. Chem. Int. Ed. Engl.* **34**, 1555–1573.

[bb2] Braga, D. & Grepioni, F. (2000). *Acc. Chem. Res.* **33**, 601–608. 10.1021/ar990143u 10995197

[bb3] Bruker (2007). *APEX2* and *SAINT* Bruker AXS Inc., Madison, Wisconsin, USA.

[bb4] Bruker (2008). *SADABS* Bruker AXS Inc., Madison, Wisconsin, USA.

[bb5] Etter, M. C. (1990). *Acc. Chem. Res.* **23**, 120–126.

[bb6] Fabbiani, P. A. F., Levendis, C. D., Buth, G., Kuhs, F. W., Shanklandd, N. & Sowa, H. (2010). *CrystEngComm*, **12**, 2354–2360.

[bb7] Loeb, S. J., Tiburcio, J., Vella, S. J. & Wisner, J. A. (2006). *Org. Biomol. Chem.* **4**, 667–680. 10.1039/b514528g 16467941

[bb8] Salitros, I., Pavlik, J., Boca, R., Fuhr, O., Rajaduraia, C. & Ruben, M. (2010). *CrystEngComm*, **12**, 2361–2368.

[bb9] Schultheis, N., Bethune, S. & Henck, J. O. (2010). *CrystEngComm*, **12**, 2436–2442.

[bb10] Sheldrick, G. M. (2008). *Acta Cryst.* A**64**, 112–122. 10.1107/S0108767307043930 18156677

